# (1*R*,6*R*,13*R*,18*R*)-(*Z*,*Z*)-1,18-Bis[(4*R*)-2,2-dimethyl-1,3-dioxolan-4-yl]-3,16-dimethyl­ene-8,20-diaza­dispiro­[5.6.5.6]tetra­cosa-7,19-diene

**DOI:** 10.1107/S1600536810023998

**Published:** 2010-06-26

**Authors:** Stéphanie M. Guéret, Peter D. W. Boyd, Margaret A. Brimble

**Affiliations:** a Department of Chemistry, The University of Auckland, Private Bag 92019, Auckland, New Zealand

## Abstract

The crystal structure of the title compound, C_34_H_54_N_2_O_4_, has been solved in order to prove the relative and absolute chirality of the newly-formed stereocentres which were established using an asymmetric Diels–Alder reaction at an earlier stage in the synthesis. This unprecedented stable dialdimine contains a 14-membered ring and was obtained as the minor diastereoisomer in the Diels–Alder reaction. The absolute stereochemistry of the stereocentres of the acetal functionality was known to be *R* based on the use of a chiral (*R*)-tris­ubstituted dienophile derived from enanti­opure (*S*)-glyceraldehyde. The assignment of the configuration in the dienophile and the title di-aldimine differs from (*S*)-glyceraldehyde due to a change in the priority order of the substituents. The crystal structure establishes the presence of six stereocentres all attributed to be *R*. The 14-membered ring contains two aldimine bonds [C—N = 1.258 (2) and 1.259 (2) Å]. It adopts a similar conformation to that proposed for *trans–trans*-cyclo­tetra­deca-1,8-dienes.

## Related literature

For related structures, see: Allmann (1974 ▶); Dale (1966 ▶). For background to the spiro­lide family, see: Gill *et al.* (2003 ▶); Guéret & Brimble (2010 ▶); Hu *et al.* (1995 ▶, 2001 ▶). For the applications of Danishefsky’s diene, see: Asano *et al.* (2006 ▶); Danishefsky *et al.* (1990 ▶); Petrzilka & Grayson (1981 ▶).
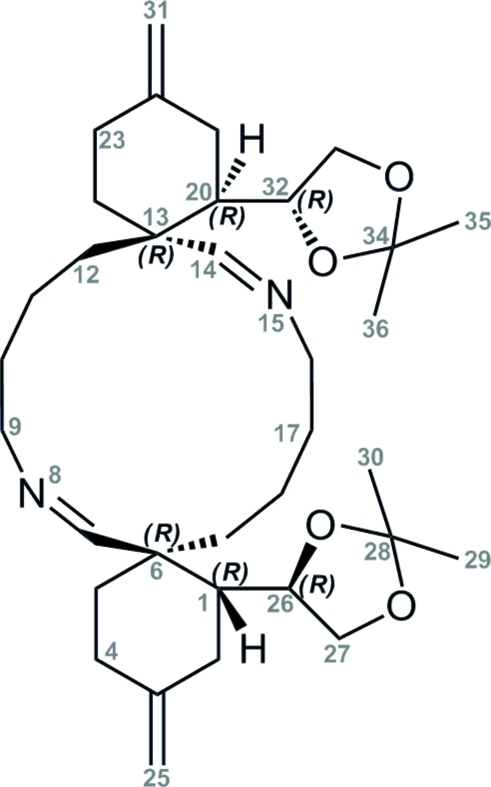


## Experimental

### 

#### Crystal data

C_34_H_54_N_2_O_4_
*M* *_r_* = 554.80
Triclinic, 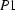

*a* = 6.8710 (1) Å
*b* = 10.1701 (2) Å
*c* = 11.7947 (2) Å
α = 79.143 (1)°
β = 88.043 (1)°
γ = 83.855 (1)°
*V* = 804.71 (2) Å^3^
*Z* = 1
Mo *K*α radiation
μ = 0.07 mm^−1^
*T* = 93 K
0.36 × 0.19 × 0.1 mm

#### Data collection

Siemens SMART CCD diffractometer
19146 measured reflections
3824 independent reflections
3555 reflections with *I* > 2σ(*I*)
*R*_int_ = 0.042

#### Refinement

*R*[*F* ^2^ > 2σ(*F* ^2^)] = 0.035
*wR*(*F* ^2^) = 0.091
*S* = 0.92
3824 reflections
365 parameters
3 restraints
H-atom parameters constrained
Δρ_max_ = 0.28 e Å^−3^
Δρ_min_ = −0.18 e Å^−3^

### 

Data collection: *SMART* (Siemens, 1995 ▶); cell refinement: *SAINT* (Siemens, 1995 ▶); data reduction: *SAINT*; program(s) used to solve structure: *SHELXS97* (Sheldrick, 2008 ▶); program(s) used to refine structure: *SHELXL97* (Sheldrick, 2008 ▶); molecular graphics: *ORTEPIII* (Burnett & Johnson, 1996 ▶), *ORTEP-3 for Windows* (Farrugia, 1997 ▶) and *Mercury* (Macrae *et al.*, 2006 ▶); software used to prepare material for publication: *WinGX* (Farrugia, 1999 ▶) and *publCIF* (Westrip, 2010 ▶).

## Supplementary Material

Crystal structure: contains datablocks I, global. DOI: 10.1107/S1600536810023998/dn2578sup1.cif

Structure factors: contains datablocks I. DOI: 10.1107/S1600536810023998/dn2578Isup2.hkl

Additional supplementary materials:  crystallographic information; 3D view; checkCIF report

## Figures and Tables

**Fig. 1.**
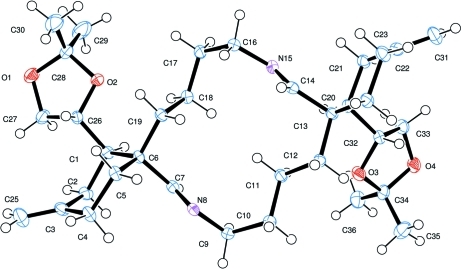
The molecular view of the title compound with the atom-labelling scheme. Displacement ellipsoids are drawn at the 50% probability level. H atoms are represented as small spheres of arbitrary radii.

**Fig. 2.**
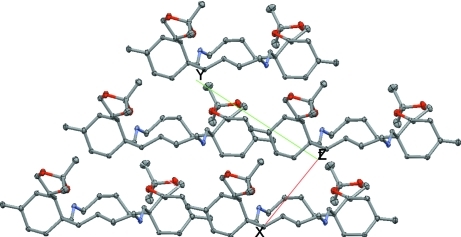
The molecular packing diagram of the title compound, viewed along c axis. The hydrogen atoms have been omitted for clarity.

## supplementary crystallographic information

### Comment

The title spiro-di-aldimine was obtained as part of a synthetic program directed towards the synthesis of the spiroimine unit of the spirolides AD. This family of marine toxins were isolated from the digestive glands of contaminated mussels, scallops and toxic plankton from the East coast of Nova Scotia in Canada and are considered as fast-acting toxins (Hu *et al.*, 1995; Hu *et al.*, 2001; Gill *et al.*, 2003; Guéret & Brimble, 2010). The work demonstrates a new method to access an enantiopure spiro-di-aldimine and an enantiopure bicyclic ketimine in good overall yield. The synthesis of the spiroimine is a synthetic challenge and to date the synthesis of the 7,6-spiroimine moiety of the spirolides has not been achieved. By reaction of a chiral (*R*)-trisubstituted dienophile derived from (*S*)-glyceraldehyde with Danishefsky's diene (Asano *et al.*, 2006; Danishefsky *et al.*, 1990; Petrzilka & Grayson, 1981), the resultant Diels-Alder adducts were afforded as a mixture of 3 diastereoisomers in a 5:2:1 ratio. The undesired minor diastereoisomer was used to develop the synthetic route to the desired spiroaldimine. The Diels-Alder adduct was converted to the spiroimine precursor in several steps. Reaction of this advanced azido-aldehyde intermediate with triphenylphosphine surprisingly afforded the stable title dimer instead of the expected 7,6-bicyclic aldimine. The stability of the title dimer is unexpected compared to the known instability of aldimines in general. Given that the stereochemistry at C26 and C32 is known to be *R* (based on using enantiopure (*S*)-glyceraldehyde as the starting material), the absolute configuration at C1, C6, C13 and C20 has therefore also been assigned as *R*. The assignment of configuration of the trisubstituted dienophile and the title di-aldimine differs from the starting (*S*)-glyceraldehyde due to a change in the priority order of substituents.

The molecular structure, Fig. 1, indicates that the acetal unit and the imine part adopt an axial position in both cyclohexane rings. The 14-membered ring contains two aldimine bonds(C14—N15 1.258 (2),C7—N8 1.259 (2)). It adopts a similar conformation to that proposed for *trans-trans* cyclotetradeca-1,8-dienes (Dale, 1966) except for an alternate conformation for C17, C18 and C19. A 14-membered *tetra*-azacyclotetradeca-1,8-diene which has *R* and *S* centres shows similar conformational characteristics (Allmann, 1974). The diazaspirocyclotetradecan-7,14-ene molecules assemble in the crystal in linear arrays. Each ring is offset with the six membered rings from a neighbouring molecule aligned over the ring centre, Fig. 2.

### Experimental

To 2-(2",2"-dimethyl-1",3"-dioxolan-4"-yl)-4-methylene-1-(4'-azidobutyl)cyclohexane carbaldehyde (7.8 mg, 24 µmol) in toluene-d8 (0.6 ml) was added triphenylphosphine (6.3 mg, 24 µmol). The resulting mixture was stirred for 1 h at room temperature then warmed to 55 °C and stirred at this temperature for 17 h. After cooling to room temperature, the mixture was concentrated in vacuo. The crude imine was purified by column chromatography (20: 80 EtOAc–hexanes) to give the *title compound* (4.6 mg, 71%) as a white crystalline solid. Dilution in CH_2_Cl_2_/hexanes (1: 1, 2 ml) and slow evaporation of the solvent afforded white prisms.

M. P. 171.8–172.3 °C.

HRMS (ESI) calculated for C_34_H_55_N_2_O_4_ [*M* + *H*]^+^: 555.4156, found 555.4143.

IR (neat) ν_max_ 3060, 2985, 2935, 1675, 1635, 1610, 1455, 1380, 1195, 1065, 895 cm^-1^.

^1^H NMR (400 MHz, CDCl_3_) *δ* 7.49 (2*H*, s, 7 and 14-C*H*=N), 4.60 (4 H, d,*J* = 28 Hz, 25 and 31-C*H*_2_=C), 4.10 (4 H, m, 26 and 32-C*H* and 27 and 33-C*H*_a_H_b_), 3.60 (2 H, m, 27 and 33-CH_a_*H*_b_), 3.47 (2 H, m, 9 and 16-C*H*_a_H_b_), 3.38 (2 H, m, 9 and 16-CH_a_*H*_b_), 2.23 (2 H, dd, *J* = 4 and 12 Hz, 2 and 21-C*H*_a_H_b_), 2.11 (6 H, t, *J* = 4 Hz, 1 and 20-C*H* and 4 and 23-C*H*_2_), 1.94 (2 H, dd, *J* = 8 and 12 Hz, 2 and 21-CH_a_*H*_b_), 1.71 (6 H, m, 5 and 24-C*H*_2_ and 12 and 19-C*H*_a_H_b_), 1.61 (4 H, m, 12 and 19-CH_a_*H*_b_ and 10 and 17-C*H*_a_H_b_), 1.45 (2 H, td, *J* = 4 and 12 Hz, 10 and 17-CH_a_*H*_b_), 1.34 (6 H, s, 29 or 30-C*H*_3_ and 35 or 36-C*H*_3_), 1.34 (6 H, s, 29 or 30-C*H*_3 _and 35 or 36-C*H*_3_), 1.22 (2 H, m, 11 and 18-C*H*_a_H_b_), 1.09 (11 and 18-CH_a_*H*_b_).

^13^C NMR (100 MHz, CDCl_3_) δ 171.8 (7 and 14-*C*H=N), 146.7 (3 and 22-*C*), 108.4 (28 and 34-*C*), 108.1 (25 and 31-*C*H_2_), 76.1 (26 and 32-*C*HO), 68.6 (27 and 33-*C*H_2_O), 60.9 (9 and 16-*C*H_2_N), 45.5 (6 and 25-*C*), 44.6 (1 and 20-*C*H), 33.4 (12 and 19-*C*H_2_), 33.2 (5 and 24-*C*H_2_), 33.1 (2 and 21-*C*H_2_), 30.9 (4 and 23-*C*H_2_), 29.9 (10 and 17-*C*H_2_), 26.7 (29 or 30-*C*H_3_ and 35 or 36-*C*H_3_), 26,3 (29 or 30-*C*H_3_ and 35 or 36-*C*H_3_), 21.5 (11 and 18-*C*H_2_).

*m/z* (ESI-MS) 195 ([*M*]^+^, 100), 278 (40), 220 (12%).

[α]_D_^20^ -25.5 (c 1/5, CH_2_Cl_2_).

### Refinement

In the absence of significant anomalous scattering, the absolute configuration could not be reliably determined from the X-ray analyses and then the Friedel pairs were merged and any references to the Flack parameter were removed.

Atoms were placed in calculated positions and a riding model (C–H = 0.93 or 0.97 Å), with *U*_iso_(H) = 1.2 or 1.5 times *U*_eq_(C) was used during refinement.

### Crystal data

**Table:**

C_34_H_54_N_2_O_4_	*Z* = 1
*M**_r_* = 554.80	*F*(000) = 304
Triclinic, *P*1	*D*_x_ = 1.145 Mg m^−^^3^
Hall symbol: P 1	Mo *K*α radiation, λ = 0.71073 Å
*a* = 6.8710 (1) Å	Cell parameters from 6455 reflections
*b* = 10.1701 (2) Å	θ = 1.8–27.9°
*c* = 11.7947 (2) Å	µ = 0.07 mm^−^^1^
α = 79.143 (1)°	*T* = 93 K
β = 88.043 (1)°	Needle, colourless
γ = 83.855 (1)°	0.36 × 0.19 × 0.1 mm
*V* = 804.71 (2) Å^3^	

### Data collection

**Table:**

Siemens SMART CCD diffractometer	3555 reflections with *I* > 2σ(*I*)
Radiation source: fine-focus sealed tube	*R*_int_ = 0.042
graphite	θ_max_ = 27.9°, θ_min_ = 1.8°
ω scans	*h* = −9→9
19146 measured reflections	*k* = −13→13
3824 independent reflections	*l* = −15→15

### Refinement

**Table:**

Refinement on *F*^2^	Primary atom site location: structure-invariant direct methods
Least-squares matrix: full	Secondary atom site location: difference Fourier map
*R*[*F*^2^ > 2σ(*F*^2^)] = 0.035	Hydrogen site location: inferred from neighbouring sites
*wR*(*F*^2^) = 0.091	H-atom parameters constrained
*S* = 0.92	*w* = 1/[σ^2^(*F*_o_^2^) + (0.0645*P*)^2^ + 0.1019*P*] where *P* = (*F*_o_^2^ + 2*F*_c_^2^)/3
3824 reflections	(Δ/σ)_max_ < 0.001
365 parameters	Δρ_max_ = 0.28 e Å^−^^3^
3 restraints	Δρ_min_ = −0.18 e Å^−^^3^

### Special details

**Table:**

Geometry. All e.s.d.'s (except the e.s.d. in the dihedral angle between two l.s. planes) are estimated using the full covariance matrix. The cell e.s.d.'s are taken into account individually in the estimation of e.s.d.'s in distances, angles and torsion angles; correlations between e.s.d.'s in cell parameters are only used when they are defined by crystal symmetry. An approximate (isotropic) treatment of cell e.s.d.'s is used for estimating e.s.d.'s involving l.s. planes.
Refinement. Refinement of *F*^2^ against ALL reflections. The weighted *R*-factor *wR* and goodness of fit *S* are based on *F*^2^, conventional *R*-factors *R* are based on *F*, with *F* set to zero for negative *F*^2^. The threshold expression of *F*^2^ > σ(*F*^2^) is used only for calculating *R*-factors(gt) *etc*. and is not relevant to the choice of reflections for refinement. *R*-factors based on *F*^2^ are statistically about twice as large as those based on *F*, and *R*- factors based on ALL data will be even larger. Three restraints for a floating origins were used.

### Fractional atomic coordinates and isotropic or equivalent isotropic displacement parameters (Å^2^)

**Table:**

	*x*	*y*	*z*	*U*_iso_*/*U*_eq_	
C5	0.4859 (3)	−0.46661 (18)	0.09602 (16)	0.0179 (3)	
H1A	0.6114	−0.4649	0.0558	0.021*	
H1B	0.5112	−0.4882	0.1782	0.021*	
C4	0.3776 (3)	−0.57809 (18)	0.06285 (17)	0.0213 (4)	
H2A	0.3781	−0.5684	−0.0206	0.026*	
H2B	0.4463	−0.6650	0.0942	0.026*	
C3	0.1690 (3)	−0.57332 (19)	0.10718 (17)	0.0220 (4)	
C2	0.0573 (3)	−0.43578 (19)	0.07628 (17)	0.0203 (4)	
H4A	−0.0731	−0.4383	0.1103	0.024*	
H4B	0.0441	−0.4122	−0.0069	0.024*	
C1	0.1606 (3)	−0.32679 (18)	0.11917 (16)	0.0157 (3)	
H5	0.0887	−0.2394	0.0889	0.019*	
C6	0.3720 (3)	−0.32518 (17)	0.06741 (15)	0.0154 (3)	
C19	0.4921 (3)	−0.22460 (17)	0.11200 (15)	0.0168 (3)	
H7A	0.5030	−0.2515	0.1951	0.020*	
H7B	0.6233	−0.2327	0.0793	0.020*	
C18	0.4100 (3)	−0.07590 (18)	0.08484 (16)	0.0181 (4)	
H8A	0.4276	−0.0419	0.0030	0.022*	
H8B	0.2705	−0.0689	0.1019	0.022*	
C17	0.5083 (3)	0.01164 (19)	0.15356 (16)	0.0217 (4)	
H9A	0.4320	0.0988	0.1453	0.026*	
H9B	0.5053	−0.0297	0.2346	0.026*	
C16	0.7199 (3)	0.03345 (19)	0.11786 (16)	0.0204 (4)	
H10A	0.7725	0.0862	0.1679	0.024*	
H10B	0.7985	−0.0529	0.1267	0.024*	
C14	0.8639 (3)	0.05893 (18)	−0.06562 (16)	0.0172 (3)	
H11	0.9483	−0.0144	−0.0314	0.021*	
C13	0.8986 (3)	0.11417 (17)	−0.19270 (15)	0.0158 (3)	
C12	0.8646 (3)	−0.00132 (17)	−0.25696 (15)	0.0166 (3)	
H13A	0.8760	0.0318	−0.3393	0.020*	
H13B	0.9680	−0.0739	−0.2363	0.020*	
C11	0.6682 (3)	−0.05878 (18)	−0.23246 (16)	0.0185 (4)	
H14A	0.5636	0.0120	−0.2564	0.022*	
H14B	0.6535	−0.0900	−0.1500	0.022*	
C10	0.6486 (3)	−0.17543 (19)	−0.29504 (16)	0.0211 (4)	
H15A	0.6386	−0.1407	−0.3773	0.025*	
H15B	0.7658	−0.2383	−0.2827	0.025*	
C9	0.4705 (3)	−0.25019 (19)	−0.25415 (16)	0.0200 (4)	
H16A	0.3527	−0.1878	−0.2649	0.024*	
H16B	0.4596	−0.3191	−0.2997	0.024*	
C7	0.3587 (3)	−0.27786 (17)	−0.06275 (15)	0.0168 (3)	
H17	0.2497	−0.2210	−0.0922	0.020*	
C24	1.1175 (3)	0.13759 (19)	−0.20847 (16)	0.0186 (4)	
H24A	1.1966	0.0552	−0.1758	0.022*	
H24B	1.1485	0.1584	−0.2903	0.022*	
C23	1.1716 (3)	0.2520 (2)	−0.15120 (18)	0.0228 (4)	
H19A	1.1577	0.2268	−0.0680	0.027*	
H19B	1.3070	0.2674	−0.1692	0.027*	
C22	1.0408 (3)	0.37918 (19)	−0.19363 (17)	0.0215 (4)	
C21	0.8255 (3)	0.36185 (18)	−0.17768 (16)	0.0192 (4)	
H21A	0.7500	0.4456	−0.2108	0.023*	
H21B	0.7945	0.3424	−0.0958	0.023*	
C20	0.7648 (3)	0.24717 (17)	−0.23484 (15)	0.0161 (3)	
H22	0.6311	0.2313	−0.2085	0.019*	
C25	0.0869 (4)	−0.6775 (2)	0.1672 (2)	0.0326 (5)	
H23A	0.1591	−0.7612	0.1848	0.039*	
H23B	−0.0430	−0.6667	0.1917	0.039*	
C26	0.1524 (3)	−0.34631 (18)	0.25092 (15)	0.0177 (3)	
H24	0.2561	−0.4151	0.2841	0.021*	
C27	−0.0430 (3)	−0.3762 (2)	0.30981 (17)	0.0253 (4)	
H25A	−0.1517	−0.3269	0.2644	0.030*	
H25B	−0.0578	−0.4716	0.3225	0.030*	
C28	0.0987 (3)	−0.2278 (2)	0.39969 (16)	0.0252 (4)	
C29	−0.0117 (5)	−0.0934 (3)	0.4064 (2)	0.0448 (7)	
H27A	−0.0607	−0.0939	0.4837	0.067*	
H27B	−0.1193	−0.0765	0.3540	0.067*	
H27C	0.0743	−0.0240	0.3858	0.067*	
C30	0.2597 (4)	−0.2692 (3)	0.4873 (2)	0.0409 (6)	
H28A	0.3296	−0.3527	0.4758	0.061*	
H28B	0.2036	−0.2803	0.5637	0.061*	
H28C	0.3482	−0.2009	0.4780	0.061*	
C31	1.1091 (3)	0.4943 (2)	−0.2410 (2)	0.0293 (4)	
H29A	1.2434	0.4991	−0.2492	0.035*	
H29B	1.0228	0.5704	−0.2660	0.035*	
C32	0.7610 (3)	0.28862 (18)	−0.36663 (16)	0.0186 (4)	
H30	0.8924	0.2709	−0.3990	0.022*	
C33	0.6765 (3)	0.4318 (2)	−0.41643 (17)	0.0232 (4)	
H31A	0.5655	0.4606	−0.3708	0.028*	
H31B	0.7745	0.4945	−0.4207	0.028*	
C34	0.5546 (3)	0.28908 (19)	−0.52033 (16)	0.0232 (4)	
C36	0.3332 (3)	0.2966 (2)	−0.5167 (2)	0.0309 (5)	
H33A	0.2833	0.3430	−0.4564	0.046*	
H33B	0.2935	0.2072	−0.5020	0.046*	
H33C	0.2825	0.3443	−0.5895	0.046*	
C35	0.6449 (4)	0.2274 (2)	−0.61933 (19)	0.0352 (5)	
H34A	0.6040	0.1391	−0.6145	0.053*	
H34B	0.7850	0.2209	−0.6152	0.053*	
H34C	0.6031	0.2829	−0.6912	0.053*	
N15	0.7312 (2)	0.10360 (16)	−0.00257 (14)	0.0188 (3)	
N8	0.4889 (2)	−0.31236 (15)	−0.13220 (13)	0.0183 (3)	
O1	−0.0310 (2)	−0.33110 (17)	0.41672 (13)	0.0323 (4)	
O2	0.1763 (2)	−0.22053 (14)	0.28513 (11)	0.0225 (3)	
O3	0.6256 (2)	0.21281 (14)	−0.41259 (12)	0.0250 (3)	
O4	0.6178 (2)	0.42036 (14)	−0.52929 (12)	0.0261 (3)	

### Atomic displacement parameters (Å^2^)

**Table:**

	*U*^11^	*U*^22^	*U*^33^	*U*^12^	*U*^13^	*U*^23^
C5	0.0147 (8)	0.0156 (8)	0.0226 (9)	−0.0016 (7)	−0.0016 (7)	−0.0012 (7)
C4	0.0204 (9)	0.0150 (8)	0.0286 (10)	−0.0041 (7)	0.0015 (7)	−0.0036 (7)
C3	0.0213 (10)	0.0216 (9)	0.0257 (10)	−0.0065 (7)	−0.0005 (8)	−0.0081 (7)
C2	0.0145 (8)	0.0245 (9)	0.0235 (9)	−0.0059 (7)	−0.0016 (7)	−0.0066 (7)
C1	0.0120 (8)	0.0160 (7)	0.0190 (8)	−0.0015 (6)	−0.0016 (6)	−0.0026 (6)
C6	0.0138 (8)	0.0144 (8)	0.0181 (8)	−0.0028 (6)	−0.0007 (6)	−0.0023 (6)
C19	0.0152 (8)	0.0162 (8)	0.0191 (8)	−0.0026 (7)	−0.0017 (7)	−0.0029 (7)
C18	0.0173 (9)	0.0176 (8)	0.0189 (8)	−0.0023 (7)	0.0006 (7)	−0.0022 (7)
C17	0.0286 (10)	0.0175 (9)	0.0202 (9)	−0.0065 (7)	0.0057 (8)	−0.0050 (7)
C16	0.0259 (10)	0.0197 (8)	0.0172 (8)	−0.0076 (7)	0.0013 (7)	−0.0048 (7)
C14	0.0165 (9)	0.0162 (8)	0.0196 (8)	−0.0024 (7)	−0.0028 (7)	−0.0044 (7)
C13	0.0152 (9)	0.0155 (8)	0.0177 (8)	−0.0030 (6)	0.0009 (7)	−0.0049 (6)
C12	0.0167 (8)	0.0153 (8)	0.0179 (8)	−0.0023 (7)	0.0021 (7)	−0.0036 (6)
C11	0.0191 (9)	0.0166 (8)	0.0206 (8)	−0.0039 (7)	0.0023 (7)	−0.0046 (7)
C10	0.0237 (9)	0.0220 (9)	0.0198 (8)	−0.0078 (7)	0.0043 (7)	−0.0075 (7)
C9	0.0239 (9)	0.0202 (8)	0.0182 (8)	−0.0075 (7)	0.0004 (7)	−0.0064 (7)
C7	0.0163 (9)	0.0141 (8)	0.0199 (8)	−0.0021 (7)	−0.0027 (7)	−0.0020 (6)
C24	0.0131 (8)	0.0202 (9)	0.0230 (9)	−0.0020 (7)	0.0022 (7)	−0.0052 (7)
C23	0.0162 (9)	0.0232 (9)	0.0301 (10)	−0.0050 (7)	0.0022 (8)	−0.0068 (8)
C22	0.0226 (10)	0.0216 (9)	0.0226 (9)	−0.0061 (8)	0.0028 (7)	−0.0087 (7)
C21	0.0209 (9)	0.0147 (8)	0.0223 (9)	−0.0023 (7)	0.0024 (7)	−0.0046 (7)
C20	0.0145 (8)	0.0156 (8)	0.0180 (8)	−0.0030 (6)	0.0024 (6)	−0.0022 (6)
C25	0.0293 (11)	0.0237 (10)	0.0451 (13)	−0.0077 (8)	0.0085 (10)	−0.0061 (9)
C26	0.0191 (9)	0.0159 (8)	0.0176 (8)	−0.0039 (7)	−0.0003 (7)	−0.0008 (6)
C27	0.0243 (10)	0.0339 (11)	0.0185 (9)	−0.0090 (8)	0.0015 (8)	−0.0040 (8)
C28	0.0303 (11)	0.0298 (10)	0.0159 (8)	−0.0079 (8)	0.0021 (8)	−0.0032 (7)
C29	0.0646 (18)	0.0409 (13)	0.0276 (11)	0.0057 (12)	0.0111 (11)	−0.0115 (10)
C30	0.0420 (14)	0.0545 (15)	0.0261 (11)	−0.0148 (12)	−0.0092 (10)	0.0001 (10)
C31	0.0250 (10)	0.0236 (10)	0.0409 (12)	−0.0096 (8)	0.0057 (9)	−0.0073 (9)
C32	0.0189 (9)	0.0162 (8)	0.0205 (8)	−0.0044 (7)	0.0006 (7)	−0.0015 (6)
C33	0.0249 (10)	0.0201 (9)	0.0234 (9)	−0.0025 (8)	−0.0019 (8)	−0.0008 (7)
C34	0.0277 (10)	0.0212 (9)	0.0186 (9)	−0.0031 (8)	−0.0005 (8)	0.0025 (7)
C36	0.0266 (11)	0.0340 (11)	0.0306 (11)	−0.0048 (9)	−0.0026 (9)	−0.0005 (9)
C35	0.0470 (14)	0.0316 (11)	0.0243 (10)	0.0027 (10)	0.0038 (10)	−0.0028 (9)
N15	0.0205 (8)	0.0176 (7)	0.0186 (7)	−0.0051 (6)	0.0020 (6)	−0.0026 (6)
N8	0.0202 (8)	0.0151 (7)	0.0209 (7)	−0.0064 (6)	0.0011 (6)	−0.0044 (6)
O1	0.0334 (9)	0.0466 (10)	0.0200 (7)	−0.0171 (7)	0.0064 (6)	−0.0085 (7)
O2	0.0295 (8)	0.0223 (6)	0.0174 (6)	−0.0072 (6)	0.0051 (5)	−0.0067 (5)
O3	0.0331 (8)	0.0200 (6)	0.0213 (7)	−0.0086 (6)	−0.0082 (6)	0.0024 (5)
O4	0.0322 (8)	0.0218 (7)	0.0220 (7)	−0.0065 (6)	−0.0028 (6)	0.0039 (5)

### Geometric parameters (Å, °)

**Table:**

C5—C4	1.536 (3)	C24—C23	1.535 (3)
C5—C6	1.546 (2)	C24—H24A	0.9700
C5—H1A	0.9700	C24—H24B	0.9700
C5—H1B	0.9700	C23—C22	1.506 (3)
C4—C3	1.507 (3)	C23—H19A	0.9700
C4—H2A	0.9700	C23—H19B	0.9700
C4—H2B	0.9700	C22—C31	1.326 (3)
C3—C25	1.327 (3)	C22—C21	1.509 (3)
C3—C2	1.509 (3)	C21—C20	1.552 (2)
C2—C1	1.547 (2)	C21—H21A	0.9700
C2—H4A	0.9700	C21—H21B	0.9700
C2—H4B	0.9700	C20—C32	1.532 (3)
C1—C26	1.529 (2)	C20—H22	0.9800
C1—C6	1.557 (2)	C25—H23A	0.9300
C1—H5	0.9800	C25—H23B	0.9300
C6—C7	1.523 (2)	C26—O2	1.439 (2)
C6—C19	1.554 (2)	C26—C27	1.523 (3)
C19—C18	1.535 (2)	C26—H24	0.9800
C19—H7A	0.9700	C27—O1	1.428 (2)
C19—H7B	0.9700	C27—H25A	0.9700
C18—C17	1.531 (3)	C27—H25B	0.9700
C18—H8A	0.9700	C28—O2	1.427 (2)
C18—H8B	0.9700	C28—O1	1.430 (3)
C17—C16	1.527 (3)	C28—C29	1.505 (3)
C17—H9A	0.9700	C28—C30	1.511 (3)
C17—H9B	0.9700	C29—H27A	0.9600
C16—N15	1.468 (2)	C29—H27B	0.9600
C16—H10A	0.9700	C29—H27C	0.9600
C16—H10B	0.9700	C30—H28A	0.9600
C14—N15	1.258 (2)	C30—H28B	0.9600
C14—C13	1.518 (2)	C30—H28C	0.9600
C14—H11	0.9300	C31—H29A	0.9300
C13—C24	1.547 (3)	C31—H29B	0.9300
C13—C12	1.554 (2)	C32—O3	1.449 (2)
C13—C20	1.560 (2)	C32—C33	1.524 (3)
C12—C11	1.525 (3)	C32—H30	0.9800
C12—H13A	0.9700	C33—O4	1.433 (2)
C12—H13B	0.9700	C33—H31A	0.9700
C11—C10	1.530 (2)	C33—H31B	0.9700
C11—H14A	0.9700	C34—O4	1.432 (2)
C11—H14B	0.9700	C34—O3	1.430 (2)
C10—C9	1.526 (3)	C34—C36	1.514 (3)
C10—H15A	0.9700	C34—C35	1.512 (3)
C10—H15B	0.9700	C36—H33A	0.9600
C9—N8	1.462 (2)	C36—H33B	0.9600
C9—H16A	0.9700	C36—H33C	0.9600
C9—H16B	0.9700	C35—H34A	0.9600
C7—N8	1.259 (2)	C35—H34B	0.9600
C7—H17	0.9300	C35—H34C	0.9600
			
C4—C5—C6	113.61 (15)	C23—C24—H24B	109.0
C4—C5—H1A	108.8	C13—C24—H24B	109.0
C6—C5—H1A	108.8	H24A—C24—H24B	107.8
C4—C5—H1B	108.8	C22—C23—C24	110.26 (16)
C6—C5—H1B	108.8	C22—C23—H19A	109.6
H1A—C5—H1B	107.7	C24—C23—H19A	109.6
C3—C4—C5	112.00 (16)	C22—C23—H19B	109.6
C3—C4—H2A	109.2	C24—C23—H19B	109.6
C5—C4—H2A	109.2	H19A—C23—H19B	108.1
C3—C4—H2B	109.2	C31—C22—C23	122.99 (19)
C5—C4—H2B	109.2	C31—C22—C21	123.65 (19)
H2A—C4—H2B	107.9	C23—C22—C21	113.35 (16)
C25—C3—C4	124.92 (19)	C22—C21—C20	112.61 (15)
C25—C3—C2	121.83 (19)	C22—C21—H21A	109.1
C4—C3—C2	113.25 (16)	C20—C21—H21A	109.1
C3—C2—C1	111.92 (15)	C22—C21—H21B	109.1
C3—C2—H4A	109.2	C20—C21—H21B	109.1
C1—C2—H4A	109.2	H21A—C21—H21B	107.8
C3—C2—H4B	109.2	C32—C20—C21	111.26 (15)
C1—C2—H4B	109.2	C32—C20—C13	113.04 (14)
H4A—C2—H4B	107.9	C21—C20—C13	110.28 (14)
C26—C1—C2	110.97 (15)	C32—C20—H22	107.3
C26—C1—C6	113.77 (14)	C21—C20—H22	107.3
C2—C1—C6	109.36 (14)	C13—C20—H22	107.3
C26—C1—H5	107.5	C3—C25—H23A	120.0
C2—C1—H5	107.5	C3—C25—H23B	120.0
C6—C1—H5	107.5	H23A—C25—H23B	120.0
C7—C6—C5	110.47 (14)	O2—C26—C27	100.21 (15)
C7—C6—C19	105.98 (14)	O2—C26—C1	109.10 (14)
C5—C6—C19	108.03 (14)	C27—C26—C1	117.03 (15)
C7—C6—C1	108.50 (14)	O2—C26—H24	110.0
C5—C6—C1	110.72 (14)	C27—C26—H24	110.0
C19—C6—C1	113.04 (14)	C1—C26—H24	110.0
C18—C19—C6	116.21 (14)	O1—C27—C26	102.98 (16)
C18—C19—H7A	108.2	O1—C27—H25A	111.2
C6—C19—H7A	108.2	C26—C27—H25A	111.2
C18—C19—H7B	108.2	O1—C27—H25B	111.2
C6—C19—H7B	108.2	C26—C27—H25B	111.2
H7A—C19—H7B	107.4	H25A—C27—H25B	109.1
C17—C18—C19	112.92 (15)	O2—C28—O1	106.40 (15)
C17—C18—H8A	109.0	O2—C28—C29	107.56 (17)
C19—C18—H8A	109.0	O1—C28—C29	110.7 (2)
C17—C18—H8B	109.0	O2—C28—C30	110.74 (19)
C19—C18—H8B	109.0	O1—C28—C30	107.89 (18)
H8A—C18—H8B	107.8	C29—C28—C30	113.4 (2)
C16—C17—C18	114.97 (15)	C28—C29—H27A	109.5
C16—C17—H9A	108.5	C28—C29—H27B	109.5
C18—C17—H9A	108.5	H27A—C29—H27B	109.5
C16—C17—H9B	108.5	C28—C29—H27C	109.5
C18—C17—H9B	108.5	H27A—C29—H27C	109.5
H9A—C17—H9B	107.5	H27B—C29—H27C	109.5
N15—C16—C17	110.75 (16)	C28—C30—H28A	109.5
N15—C16—H10A	109.5	C28—C30—H28B	109.5
C17—C16—H10A	109.5	H28A—C30—H28B	109.5
N15—C16—H10B	109.5	C28—C30—H28C	109.5
C17—C16—H10B	109.5	H28A—C30—H28C	109.5
H10A—C16—H10B	108.1	H28B—C30—H28C	109.5
N15—C14—C13	125.76 (16)	C22—C31—H29A	120.0
N15—C14—H11	117.1	C22—C31—H29B	120.0
C13—C14—H11	117.1	H29A—C31—H29B	120.0
C14—C13—C24	107.37 (14)	O3—C32—C33	100.30 (15)
C14—C13—C12	105.58 (14)	O3—C32—C20	109.32 (15)
C24—C13—C12	107.31 (14)	C33—C32—C20	117.28 (15)
C14—C13—C20	111.65 (14)	O3—C32—H30	109.8
C24—C13—C20	110.94 (14)	C33—C32—H30	109.8
C12—C13—C20	113.61 (14)	C20—C32—H30	109.8
C11—C12—C13	115.48 (15)	O4—C33—C32	102.53 (15)
C11—C12—H13A	108.4	O4—C33—H31A	111.3
C13—C12—H13A	108.4	C32—C33—H31A	111.3
C11—C12—H13B	108.4	O4—C33—H31B	111.3
C13—C12—H13B	108.4	C32—C33—H31B	111.3
H13A—C12—H13B	107.5	H31A—C33—H31B	109.2
C12—C11—C10	112.45 (15)	O4—C34—O3	106.23 (15)
C12—C11—H14A	109.1	O4—C34—C36	110.57 (17)
C10—C11—H14A	109.1	O3—C34—C36	108.03 (16)
C12—C11—H14B	109.1	O4—C34—C35	108.48 (17)
C10—C11—H14B	109.1	O3—C34—C35	110.33 (17)
H14A—C11—H14B	107.8	C36—C34—C35	112.99 (19)
C9—C10—C11	112.78 (16)	C34—C36—H33A	109.5
C9—C10—H15A	109.0	C34—C36—H33B	109.5
C11—C10—H15A	109.0	H33A—C36—H33B	109.5
C9—C10—H15B	109.0	C34—C36—H33C	109.5
C11—C10—H15B	109.0	H33A—C36—H33C	109.5
H15A—C10—H15B	107.8	H33B—C36—H33C	109.5
N8—C9—C10	110.21 (15)	C34—C35—H34A	109.5
N8—C9—H16A	109.6	C34—C35—H34B	109.5
C10—C9—H16A	109.6	H34A—C35—H34B	109.5
N8—C9—H16B	109.6	C34—C35—H34C	109.5
C10—C9—H16B	109.6	H34A—C35—H34C	109.5
H16A—C9—H16B	108.1	H34B—C35—H34C	109.5
N8—C7—C6	122.82 (16)	C14—N15—C16	117.19 (16)
N8—C7—H17	118.6	C7—N8—C9	118.06 (16)
C6—C7—H17	118.6	C27—O1—C28	107.81 (15)
C23—C24—C13	113.04 (15)	C28—O2—C26	107.24 (14)
C23—C24—H24A	109.0	C34—O3—C32	108.65 (14)
C13—C24—H24A	109.0	C34—O4—C33	107.11 (14)
